# Common Data Elements for Meaningful Stroke Documentation in Routine Care and Clinical Research: Retrospective Data Analysis

**DOI:** 10.2196/27396

**Published:** 2021-10-12

**Authors:** Sarah Berenspöhler, Jens Minnerup, Martin Dugas, Julian Varghese

**Affiliations:** 1 Institute of Medical Informatics Westfälische Wilhelms-University Münster Münster Germany; 2 Department of Neurology with Institute of Translational Neurology University Hospital Münster Münster Germany; 3 Institute of Medical Informatics Heidelberg University Hospital Heidelberg Germany

**Keywords:** common data elements, stroke, documentation

## Abstract

**Background:**

Medical information management for stroke patients is currently a very time-consuming endeavor. There are clear guidelines and procedures to treat patients having acute stroke, but it is not known how well these established practices are reflected in patient documentation.

**Objective:**

This study compares a variety of documentation processes regarding stroke. The main objective of this work is to provide an overview of the most commonly occurring medical concepts in stroke documentation and identify overlaps between different documentation contexts to allow for the definition of a core data set that could be used in potential data interfaces.

**Methods:**

Medical source documentation forms from different documentation contexts, including hospitals, clinical trials, registries, and international standards, regarding stroke treatment followed by rehabilitation were digitized in the operational data model. Each source data element was semantically annotated using the Unified Medical Language System. The concept codes were analyzed for semantic overlaps. A concept was considered common if it appeared in at least two documentation contexts. The resulting common concepts were extended with implementation details, including data types and permissible values based on frequent patterns of source data elements, using an established expert-based and semiautomatic approach.

**Results:**

In total, 3287 data elements were identified, and 1051 of these emerged as unique medical concepts. The 100 most frequent medical concepts cover 9.51% (100/1051) of all concept occurrences in stroke documentation, and the 50 most frequent concepts cover 4.75% (50/1051). A list of common data elements was implemented in different standardized machine-readable formats on a public metadata repository for interoperable reuse.

**Conclusions:**

Standardization of medical documentation is a prerequisite for data exchange as well as the transferability and reuse of data. In the long run, standardization would save time and money and extend the capabilities for which such data could be used. In the context of this work, a lack of standardization was observed regarding current information management. Free-form text fields and intricate questions complicate automated data access and transfer between institutions. This work also revealed the potential of a unified documentation process as a core data set of the 50 most frequent common data elements, accounting for 34% of the documentation in medical information management. Such a data set offers a starting point for standardized and interoperable data collection in routine care, quality management, and clinical research.

## Introduction

### Background

Stroke is the second most common cause of death worldwide and is the most important cause of permanent disability in adults [1]. Owing to the increasing aging of the population, a steady incidence rate would probably lead to an increasing number of people being affected by stroke in Germany in the next decades [1].

In addition, the treatment of the disease and the consequent damage generate immense costs for the health system and thus the population [2]. The high incidence and prevalence rate of this disease in Germany induces numerous studies, the creation of therapy guidelines and regulations for proper initial treatment of stroke patients, and preventive measures. Currently, there are more than 300 certified stroke units in Germany [3]. These stroke units have to comply to a multitude of certification criteria and pass regular audits [3]. Primary as well as standardized secondary prevention (including early recurrence prevention) are also of great importance, and procedures for treating carotid artery stenosis are regulated by the S3 guidelines [4].

What about information management regarding stroke patients are there sufficient standards and guidelines for hospitals as well? It is common knowledge that patient documentation is a time-consuming endeavor. The documentation already starts in the emergency room when basic patient data are collected, and the medical history and initial examination results are recorded. Subsequent examination results, vital parameters, and health changes were documented according to a fixed time schedule. Even after hospitalization, a large amount of data are recorded during follow-up examinations, rehabilitation, or clinical research. Medical documentation accounts for 25% of the physician’s workload and takes up as much time as direct patient care [5].

### Objective

Although there are clear certification requirements for stroke units, guidelines for therapy, extensive rehabilitation networks, secondary prevention, and numerous research papers, the question arises as to what measures are taken to improve the collection and processing of data. Standardization allows for a sound way of transferring data between departments and institutions, which saves time and money [6,7]. Do such standardizations or maybe even a transferable core data set already exist? Queried institutions denied questions regarding standardizations for routine clinical documentation, software, transmission interfaces, or a core data set; there is also no superordinate institution for coordinating the exchange of data. The certification criteria for stroke units specify certain examinations, examination scores, and therapy cycles; documentation is expected, but there are no specific requirements or standards.

In some federal states of Germany, the only clear requirement specific to information management concerning stroke units is mandatory participation in the stroke registry for quality assurance. Clinics must provide a form with 77 data elements for each patient and are required to complete at least 90% of these [8]. The data elements are revised annually and partially adjusted.

The association of German stroke registries, the Arbeitsgemeinschaft Deutschsprachiger Schlaganfall-Register (ADSR), is a voluntary association of regional quality assurance projects regarding stroke treatment. The ADSR was founded in 1999 with the objective of developing a standardized data collection for stroke cases. It creates regional as well as supraregional comparisons based on scientific, quality-related, and epidemiological viewpoints. There are yearly meetings for members to reconcile documentation forms and discuss uniform quality indicators. These quality indicators are developed by a multidisciplinary work group, including representatives of the Deutsche Schlaganfall Gesellschaft (German Stroke Society), the Deutsche Gesellschaft für Neurologie (German Association of Neurology) and the Stiftung Deutsche Schlaganfall-Hilfe (German Stroke Foundation). Approximately 300,000 data records are evaluated by the ADSR each year [9]. The documentation for the stroke registry is usually conducted with additional software, so data from routine documentation are not taken over. Redundant documentation performed in parallel for different applications increases the effort and susceptibility to errors.

There are also registries for quality assurance in the domain of early rehabilitation and rehabilitation in general. The Hessische Krankenhausgesellschaft (Hesse Hospital Association) and health insurance associations in Hesse have come to a contractual agreement in this regard, but supraregionally there is no obligation to participate.

Problems with existing documentation procedures are not new. For many years, medical information management has been analyzed and discussed in the field of health informatics. Thus, studies similar to this one exist for other diseases, such as for acute myeloid leukemia [10] or acute coronary syndrome [11]. Since 2015, the German Research Foundation has funded several projects that aim to establish an information infrastructure for research data. In this context, more than 500,000 additional data models were processed at the Institute of Medical Informatics in Münster [12].

So far, endeavors regarding information management for stroke patients in Germany have been covered, but what is the international situation with regard to this? Are there standards, interfaces for different medical documentation sources, or even a specification of a core data set? The National Institute of Neurological Disorders and Stroke (NINDS) is a research institute belonging to the National Institutes of Health and is supported by the Department of Health and Human Services. One of the objectives of the NINDS is to develop data standards for clinical research and accessible tools while improving data quality and cost control [13,14]. Data elements from case report forms, clinical routine forms, guidelines, and clinical data standards are available to identify core data elements [13,15-17].

There are also registries for patients who had stroke in other countries. Thus, data structures from the Austrian Stroke Registry were included in this study [18].

In summary, the objective of this work is to contribute to a crossdomain core data set for patients who had stroke that could function as a standard and be used for data exchange.

## Methods

### Overview of the Methods

**Figure 1:**
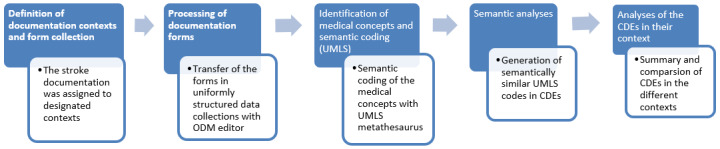
Overall workflow for definition of CDEs from existing documentation forms. CDE: common data element; ODM: operational data monitor; UMLS: Unified Medical Language System.

Figure 1 illustrates the conducted main steps from the definition of documentation contexts over transfer of uniformly structured data and semantic coding to create and comparison of common data elements’ (CDEs).

### Definition of Documentation Contexts and Form Collection

**Figure 2:**
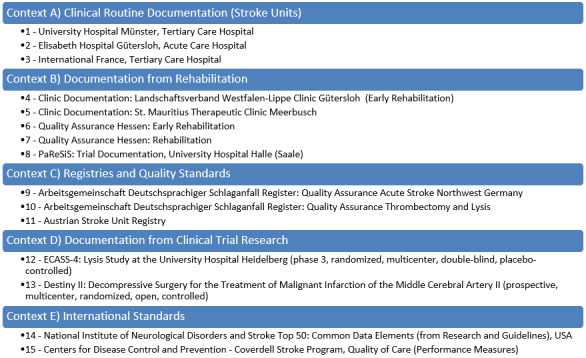
Designated contexts. ADSR: Arbeitsgemeinschaft Deutschsprachiger Schlaganfall-Register; CDC: Centers for Disease Control and Prevention; ECASS-4: European Cooperative Acute Stroke Study-4; NINDS: National Institute of Neurological Disorders and Stroke.

Patient documentation forms for stroke patients were collected from hospitals, rehabilitation facilities, research papers, registries, and standards for the period from 2014 to 2017 (Figure 2). The selection was conducted by 2 medical experts including 1 clinical stroke expert based on the availability of documentation forms and broad content coverage in clinical research and care.

Documentation forms were collected from different contexts to provide a broad landscape of clinical care and clinical research documentation. The process of form collection and ensuring broad coverage by different documentation contexts is based on an established approach for CDE generation in the field of acute myeloid leukemia [10] and acute coronary syndrome [11]. Core contexts include routine clinical documentation, trial and register documentation, and international standards. To regard stroke-specific documentation, the context *rehabilitation* was added, as this was considered a highly relevant part of follow-up stroke care.

The core contexts of this work as follows:

1. Clinical routine documentation (stroke units): For this work the clinical routine documentation of the University Hospital Münster as a tertiary care hospital and the Elisabeth Hospital Gütersloh as an acute care hospital is considered, as well as the documentation from a tertiary care hospital in France. All forms were selected and made available by specialists working at these hospitals.
2. Documentation from rehabilitation: this category contains the clinical routine documentation from a facility for early rehabilitation in Gütersloh, a rehabilitation clinic in Meerbusch, a registry for early [19] and general rehabilitation [20] and also trial documentation from the University Hospital Halle [21].
3. Registries and quality standards: Two data models from the association of German stroke registries (ADSR) are considered, one regarding acute care for stroke patients in hospitals and one regarding thrombectomy and lysis therapy after stroke [9,22]. Moreover, a registry from Austria was included in the analysis [18].
4. Documentation from clinical trial research: The University Hospital Heidelberg provided the documentation for two clinical trials, a phase 3 trial researching lysis therapy for stroke patients (parameters: randomized, multicentre, double-blind, and placebo-controlled) and a trial researching decompressive surgery after severe stroke (parameters: prospective, multicentre, randomized, open, and controlled) [23,24].
5. International standards: The NINDS in the United States provides a list of the 50 most CDEs collected mainly from clinical trial documentation and guidelines [25]. Furthermore, the Centers for Disease Control and Prevention (CDC) provides performance measures for the quality of care–both of these data sets are considered in this work [26].

### Processing of Documentation Forms and Semantic Coding

The collected forms were transformed into a unified document structure, called the operational data model (ODM) by the Clinical Data Interchange Standards Consortium. To do this, forms were created using the ODM editor available on the Medical Data-Models portal [12]. This portal is a metadata registry operated by the Institute of Medical Informatics in Münster and can be used to create, analyze, share, and reuse medical forms; it serves as an infrastructure for academic medical research (noncommercial) [16]. Each data element in the ODM format was assigned a Unified Medical Language System (UMLS) concept code. The UMLS Metathesaurus [27] contains UMLS concepts and incorporates important terminology, classification, and coding standards. For instance, the data element *birth date of patient* was allocated to the concept code *C0421451 Patient Date of Birth*.

### Analysis and Generation of CDEs

UMLS-encoded ODM forms were analyzed using the CDE generator to identify common concepts and to generate CDEs [28]. The CDE generator is a publicly accessible tool and was used to count and display assigned UMLS codes ordered by frequency. It also enables the generation of cumulative concept coverage and pairwise comparisons of different documentation contexts. A concept was selected as a common concept if it appeared in at least two documentation contexts. By adding the most common information on datatypes, and permissible values from the analyzed sources, the corresponding CDEs were generated. A similar methodology was applied to other disease domains before [10,11].

Some concepts had to be aggregated and corrected to reveal overlaps between domains, for example, the concept *C0525032 International Normalized Ratio* and the concept *C0030605 Activated Partial Thromboplastin Time Measurement* were subsumed under the concept *C0005790 Blood Coagulation Tests* as part of the code harmonization. All forms and UMLS codes were checked by an experienced UMLS coder, and the resulting list of CDEs was also reviewed by a neurologist and a stroke specialist.

Finally, a list of the most frequented CDEs can be generated. In the following, the top-30 CDE extract shows the 30 most frequent concepts, which were used in at least two documentation contexts. The resulting list is shared on the Medical Data-Models portal for reuse.

## Results

### Data Collection

A total of 3287 data elements were identified based on 15 medical information management systems (Figure 2), from which 1051 unique medical concepts emerged.

### UMLS Coverage and Missing Concepts

Some data elements could not be assigned to a UMLS concept code in an unambiguous way; for instance, individual answers to intricate questions or complex instructions that were simply ticked off in a form (eg, *dose: 0.9 mg × kg body weight, maximum dose 90 mg, 10% initially as a bolus, the rest over 60 minutes via a syringe pump*). Moreover, administrative items such as dates and signatures appearing in medical reports were excluded from the concept code allocation. This study mainly focuses on medically relevant concepts.

### Cumulative Concept Coverage

**Figure 3:**
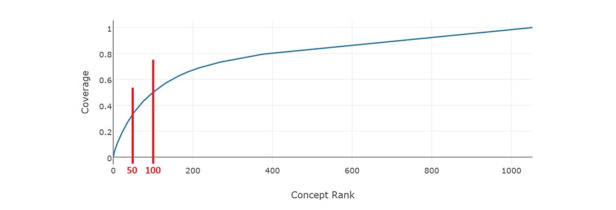
Cumulative concept coverage.

Cumulative frequencies help assess the heterogeneity of concepts. Among 1051 unique medical concepts, the 50 most frequent ones accounted for 34% of all concept occurrences in the collected stroke documentation (Figure 3). Expanding this to the 100 most frequent concepts leads to a coverage rate of 50%. The most frequent concept, *C0031809 Physical Examination*, refers to the general physical examination including the examination of the liver, the kidneys, the lungs, etc. Another frequently appearing concept (with a frequency of 43) was *C0003280 Anticoagulants* containing thrombosis prophylaxis and therapy, the dosing of heparin and vitamin K antagonists, etc; the concept *C3702515 Evaluation of Speech*, including examinations concerning articulation disorders, dysarthria, and apraxia, also appeared often. After the 363 most frequent concepts, the subsequent ones appeared only once. A complete list of concepts is provided in Multimedia Appendix 1.

### Comparison of Documentation Contexts

#### Comparison of Clinical Routine Documentation and Documentation from Registries and Quality Standards (Context A and C)

Overall, routine clinical documentation of the stroke units contained 537 unique medical concepts and 206 concepts in registries and standards. Afterwards, the domains could be compared with the CDE generator. There was an overlap of 100 concepts between the information managements of the three stroke units and the information managements of the registries and standards (3 as well, Table 1); therefore, 100 concepts appeared in both domains, for example, concepts like *C0543414 Tobacco Use* or *C0007465 Cause of Death*. In contrast, concepts such as *C0018810 Heart Rate* only appeared in the information managements of the stroke units and *C0162578 Thrombectomy* only appeared in the information management of the registries and standards.

**Table 1:** Overview of overlaps between different contexts^a^.

Contexts	Context A: clinical routine documentation (537 distinct concepts)	Context B: rehabilitation (475 distinct concepts)	Context C: registries and quality standards (206 distinct concepts)	Context D: clinical trial research (181 distinct concepts)	Context E: international standards (56 distinct concepts)
Context A: clinical routine documentation (537 distinct concepts)	*537*/100.0%/100.0%	*147*/27.4%/30.9%	*100*/18.6%/48,5%	*84*/15.6%/46.4%	*35*/6.5%/62.5%
Context B: rehabilitation (475 distinct concepts)	*147*/30.9%/27.4%	*475*/100.0%/100,0%	*85*/17.9%/41.3%	*52*/10.9%/28.7%	*28*/5.9%/50.0%
Context C: registries and quality standards (206 distinct concepts)	*100*/48.5%/18.6%	*85*/41.3%/17.9%	*206*/100.0%/100.0%	*56*/27.2%/30.9%	*25*/12.1%/44.6%
Context D: clinical trial research (181 distinct concepts)	*84*/46.4%/15.6%	*52*/28.7%/10.9%	*56*/30.9%/27.2%	*181*/100.0%/100,0%	*26*/14.4%/46.4%
Context E: international standards (56 distinct concepts)	*35*/62.5%/6.5%	*28*/50.0%/5.9%	*25*/44.6%/12.1%	*26*/46.4%/14.4%	*56*/100%/100%

^a^The first number (italicized) represents the number of common concepts between two contexts. For example, the second grid cell shows 147 shared concepts between context A and B, which corresponds to an overlap rate of 27.4% regarding context A (147/537) and 30.9% regarding context B (147/475).

#### Comparison of Clinical Routine Documentation and Documentation from Clinical Trial Research (Context A and D)

While the information management of the stroke units contained 537 unique medical concepts, the documentation from the clinical trial research (consisting of two trials, Table 1) yielded 181 unique medical concepts.

In total, 84 of these 181 concepts appeared in both the documentation from clinical trial research and clinical routine documentation, so there was an overlap of 46.4% between these domains. Examples of such overlapping concepts are *C0030193 Pain*, *C0005790 Blood Coagulation Tests,* and *C3476804 NIHSS.* Results from physical therapy (*C0949766 Physical Therapy*) were only recorded in the stroke units but did not appear in the clinical trials. By contrast, the concept *C3872877 Hemicraniotomy* was only found in the documentation from clinical trial research.

#### Comparison of National Registries and Standards (Germany) and International Standards (Context C and E)

Documentation from national registries and standards revealed 206 unique medical concepts, whereas only 56 concepts emerged from international standards. A total of 25 concepts appeared in both domains, for example *C0003811 Cardiac Arrhythmia*, *C0003280 Anticoagulants,* and *C0001948 Alcohol Consumption*. The concept *C2984908 Modified Rankin Scale* was only present in the German registries and standards and the concept *C0013658 Educational Status* only appeared in international standards.

#### International Standards and Documentation From Clinical Trial Research in Germany (Context E and D)

Documentation from two clinical trials conducted in Germany yielded 181 unique medical concepts, whereas the top list from the NINDS and the performance measures from the CDC contained 56 concepts. Of these 56 international standards concepts 26 (46%) overlapped with the concepts from the clinical trials, which represented 14.4% of the German trial documentation. Concepts like *C0005823 Blood Pressure*, *C2361123 Discharge Date,* and *C0040044 Thrombolytic Therapy* appeared in both domains, whereas the concept *C0009566 Complication* only appeared in the German clinical trials and the concept *C0001948 Alcohol Consumption* only appeared in the NINDS list of international standards.

#### Comparison of the Clinical Routine Documentation for Stroke Units and Rehabilitation Facilities (Domain A and Subdomain of B)

The routine documentation of the three stroke units contained 537 unique medical concepts, whereas the documentation of the rehabilitation clinics contained 475 unique medical concepts.

A comparison between these domains revealed an overlap of 147 concepts, for example, *C0011900 Diagnosis*, *C0154251 Lipid Metabolism Disorders,* and *C1305855 Body Mass Index.* The concept covering results from the ECG (*C0013798: Electrocardiogram*) was only present in the stroke unit documentation and *C0260682: Tracheostomy Status* and *C0233414: Disturbance of Attention* are examples of concepts that only appear in the documentation of the rehabilitation facilities.

### Overview of CDEs

A list of the 50 most frequent concepts (based on the selection of a medical expert and medical computer scientists) could be created. Table 2 shows an extract containing the top 30 and sorted to different documentation categories. The absolute frequency of a concept could exceed the number sources (n=15), as some concepts appeared more than one in a source. A model implementation for an extended list of the 50 most common concepts was created as a Clinical Data Interchange Standards Consortium–compliant implementation for reuse (attached in the Multimedia Appendix 1).

**Figure 4:**
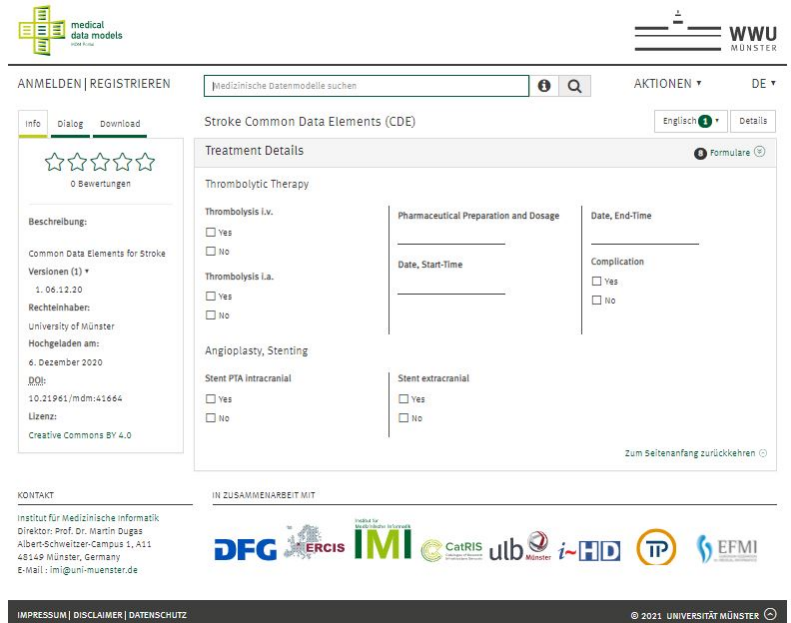
The core data set in stroke care and research is available at the MDM-portal of the Institute of Medical Informatics in Münster [29].

Moreover, the actual implementation according to the Clinical Data Interchange Standards Consortium ODM is available on the web [12] (Figure 4).

**Table 2:** Top 50 of most frequent concepts with absolute concept frequency and sorted by medical category^a^.

No.	Concept	Documentation category	ACF	A	B	C	D	E
1	Patient internal identifier	Administrative/demographics	12	✓	✓	✓	✓	
2	Patient name	Administrative/demographics	9	✓	✓	✓	✓	
3	Gender	Administrative/demographics	9	✓	✓	✓	✓	✓
4	Patient date of birth, age	Administrative/demographics	19	✓	✓	✓	✓	✓
5	Date of admission	Administrative/demographics	13	✓	✓	✓	✓	✓
6	Discharge date	Administrative/demographics	12	✓	✓	✓	✓	✓
7	Living situation (alone, independent, or family home)	Administrative/demographics	17	✓	✓	✓	✓	
8	Death (finding, date/time)	Administrative/demographics	18	✓	✓	✓	✓	
9	Symptom findings in relation to time, time to treatment	Diagnostic/medical history	23	✓		✓	✓	✓
10	Diagnosis (transient ischemic attack, Ischemic stroke and localization, brain hemorrhage, and localization)	Diagnostic/medical history	132	✓	✓	✓	✓	✓
11	History of cerebrovascular accident and further medical history	Diagnostic/medical history	21	✓	✓	✓	✓	
12	Etiology	Diagnostic/medical history	19	✓		✓	✓	
13	Pre-existing conditions and risk factors	Diagnostic/medical history	104	✓	✓	✓	✓	✓
14	Vital signs (blood pressure, heart rate, SpO2, breathing rate, body temperature)	Diagnostic/medical history	67	✓	✓	✓	✓	✓
15	Neurologic symptoms and neurological deficit	Examination/follow-up	8	✓		✓		
16	General physical examination	Examination/follow-up	65	✓	✓	✓	✓	
17	National Institutes of Health Stroke Scale	Examination/follow-up	142	✓	✓	✓	✓	✓
18	Dysphagia/deglutition disorders	Examination/follow-up	23	✓	✓	✓		✓
19	Quality of vision-vision disorders?	Examination/follow-up	13	✓	✓			
20	Modified Rankin Scale	Nursing issues, rehabilitation	25	✓	✓	✓	✓	
21	Barthel index	Nursing issues, rehabilitation	17		✓	✓	✓	
22	Diagnostic imaging (magnetic resonance imaging, computed tomography, and ultrasonography)	Apparatus-based diagnostics	73	✓	✓	✓		
23	Angiography and digital subtraction	Apparatus-based diagnostics	9	✓	✓	✓		✓
24	Routine blood tests	Laboratory: blood panel	93	✓		✓	✓	
25	Medication list	Medication	37	✓	✓	✓	✓	✓
26	Anticoagulants	Medication	43	✓	✓	✓	✓	✓
27	Antiplatelet agents	Medication	30	✓	✓	✓	✓	✓
28	Thrombolytic therapy	Treatment details	23	✓	✓	✓	✓	✓
29	Angioplasty and stenting	Treatment details	26	✓		✓		
30	Physiotherapy and ergotherapy	Treatment details	38	✓	✓	✓		

^a^The common data elements (CDEs) for physiotherapy and ergotherapy are listed together in the 30th line. Columns, A, B, C, D and E present the occurrence of CDEs in the documentations of the according contexts (findings in Figure 2: Context A: Clinical routine documentation (stroke units), Context B: Documentation from rehabilitation, Context C: Registries and quality standards, Context D: Documentation from clinical trial research, and Context E: International standards).

## Discussion

### Principal Findings

This work systematically compared data from routine care, research, and quality management to derive a core data set, which could function as a starting point for further standardization efforts. The 50 most frequent of the 3287 concepts could already cover 34% of all concepts occurring in the entire data item collection. This shows the high potential of a potential core data set. The list has been published and exported in various documentation formats. This could facilitate data exchange between different institutions

During form collection, a few factors surfaced that made it a challenging endeavor: Issues related to intellectual property and confidentiality aspects impeded the availability of documentation forms, especially that of case report forms used in clinical trial research. Moreover, the software used in hospitals and rehabilitation facilities varied quite a bit, and often data were not available in digital form.

A general problem, especially with regard to routine documentation, is the numerous free-form text fields, which are only coded by an unspecific concept. In clinical routines, medical findings are often entered as free-text, whereas specific data points are usually queried in registries and standards and clinical trial research. The information captured by specific queries was also often found in free-form text fields in routine documentation; for example, a free-form text field asking for pre-existing conditions on the one hand and checkboxes for specific conditions (such as hypertension and atrial fibrillation) on the other.

We noticed a relatively small overlap of 25 concepts between national and international standards (comparison of national registries and standards [Germany] and International Standards [Context C and E]), which could be explained by the fact that German registries and standards focus mainly on routine documentation whereas the international standards of the NINDS on clinical trial research.

Clinical trials usually try to answer specific research questions (International standards and Documentation from Clinical Trial Research in Germany [Context E and D]). There was an overlap of 26 concepts between the international standards (represented by the top list of NINDS with the performance measures of CDC) and the documentations from clinical trials in Germany, but the overlaps were limited to basic patient data such as body weight, vital signs, and gender.

An interconnected and unified solution would facilitate the exchange and reuse of data, which would bring many benefits; for example, the automatic creation of text blocks for discharge summaries would be possible. A more transparent documentation process would also simplify the efforts for standardization and quality assurance. Patient data from routine clinical documentation could be transferred to rehabilitation facilities, registries, and researchers conducting clinical trials. Data could also be reused, for instance, for further studies (secondary use), and it could easily be used for electronic health records.

This insight is not new, although earlier works and studies have already pointed out potential savings of both cost and time on the basis of CDEs for a more unified documentation process [6,7,11,30,31]. However, the implementation of CDEs and semantic annotations is not trivial. Some medical facilities and institutes would need to upgrade all their software and data entry forms that are currently still kept in paper form. There are many free-form text fields in current forms that are completed by individuals in a subjective manner. In this study, only the headers of free-form text fields were allocated to the concept codes. This led to important information not being covered, a smaller number of assigned concept codes, and thus, some overlaps are not recognizable.

UMLS is currently the most important approach for unifying the terminologies of biomedical resources, such as web-based databases and medical dictionaries. However, there are many UMLS codes that are quite similar and semantically nearly identical. Examples of such UMLS concept codes are *C2361123 Discharge Date*, *C2710998 Hospital Discharge Date*, and *C2361122 Discharge Date:Time Stamp--Date and Time:Point in Time:^Patient:Quantitative.* The coder has to decide which one to use, so encoding is a time-consuming process that is ideally performed by an experienced coder. A medical expert with experience in coding reviewed the concept of encoding for this work. The problem of uniform quality assurance for the assignment of CDEs was described previously [32,33].

### Conclusions

Standardizing data from medical information management systems is necessary to reduce the amount of work needed for patient documentation and to allow for efficient querying, transfer, and reuse of data. Currently, there are no uniform standards for data collection regarding stroke across different domains. It would be strongly advisable to create a committee or work group to harmonize research and care relevant documentation for effective data reuse. This work provides a list of harmonized common data items based on existing stroke-related documentation, which can be reused to harmonize future documentation efforts in stroke-related care or research.

## Appendix

Multimedia Appendix 1 A complete list of concepts.

## Abbreviations

ADSR: Arbeitsgemeinschaft Deutschsprachiger Schlaganfall-Register
CDC: Centers for Disease Control and Prevention
CDE: common data element
NINDS: National Institute of Neurological Disorders and Stroke
ODM: operational data model
UMLS: Unified Medical Language System
